# Ebola Virus–Specific Neutralizing Antibody Persists at High Levels in Survivors 2 Years After Resolution of Disease in a Sierra Leonean Cohort

**DOI:** 10.1093/infdis/jiae155

**Published:** 2024-05-27

**Authors:** Nell G Bond, Kayla R Shore, Emily J Engel, Erin E Coonan, Foday Al-Hasan, Michael A Gbakie, Fatima K Kamara, Lansana Kanneh, Mambu Momoh, Ibrahim M Kanneh, John D Sandi, Debra Elliott, Samuel C Ficenec, Ashley R Smira, William A Fischer, David A Wohl, James E Robinson, Jeffrey G Shaffer, Robert F Garry, Robert J Samuels, Donald S Grant, John S Schieffelin

**Affiliations:** Tulane University, New Orleans, Louisiana; Tulane University, New Orleans, Louisiana; Tulane University, New Orleans, Louisiana; Tulane University, New Orleans, Louisiana; Kenema Government Hospital, Sierra Leone; Kenema Government Hospital, Sierra Leone; Kenema Government Hospital, Sierra Leone; Kenema Government Hospital, Sierra Leone; Kenema Government Hospital, Sierra Leone; Eastern Technical University, Kenema, Sierra Leone; Kenema Government Hospital, Sierra Leone; Kenema Government Hospital, Sierra Leone; Tulane University, New Orleans, Louisiana; Tulane University, New Orleans, Louisiana; Tulane University, New Orleans, Louisiana; University of North Carolina, Chapel Hill; University of North Carolina, Chapel Hill; Tulane University, New Orleans, Louisiana; Tulane University, New Orleans, Louisiana; Tulane University, New Orleans, Louisiana; Kenema Government Hospital, Sierra Leone; Kenema Government Hospital, Sierra Leone; Tulane University, New Orleans, Louisiana

**Keywords:** antibody persistence, Ebola, Ebola virus disease survivor, unrecognized Ebola transmission, humoral immunity

## Abstract

Ebola virus (EBOV) infection results in Ebola virus disease (EVD), an often severe disease with a nonspecific presentation. Since its recognition, periodic outbreaks of EVD continue to occur in sub-Saharan Africa. The 2013–2016 West African EVD outbreak was the largest recorded, resulting in a substantial cohort of EVD survivors with persistent health complaints and variable immune responses. In this study, we characterize humoral immune responses in EVD survivors and their contacts in Eastern Sierra Leone. We found high levels of EBOV IgG in EVD survivors and lower yet substantial antibody levels in household contacts, suggesting subclinical transmission. Neutralizing antibody function was prevalent but variable in EVD survivors, raising questions about the durability of immune responses from natural infection with EBOV. Additionally, we found that certain discrete symptoms—ophthalmologic and auditory—are associated with EBOV IgG seropositivity, while an array of symptoms are associated with the presence of neutralizing antibody.

## Teaser

We measured Ebola virus–specific IgG antibody and antibody neutralization potential in a cohort of 357 Ebola virus disease survivors and 969 contacts. We found that Ebola virus IgG and antibody neutralization potential persisted in the survivor population but was variable across the cohort.

Ebola virus disease (EVD) is a severe viral hemorrhagic fever that presents nonspecifically and often progresses rapidly with hemorrhagic manifestations and ultimately death [1]. The largest Ebola virus (EBOV) outbreak began in Guinea in late 2013 [2], lasting through June 2016 with >28 600 cases and a case fatality rate of 39% [2]. Nearly half of all cases occurred in Sierra Leone, of which 28% were fatal [2]. Additional EBOV outbreaks continue to occur in West and Central Africa [1]. Two of these outbreaks were traced back to EVD survivors with no known reexposure to EBOV [3, 4]. Outbreaks over the past decade have left a large number of EVD survivors with persistent health complaints and variable sustained protective immune responses [5–8]. Considering the high virulence of EBOV and its continued emergence, it is imperative to gain further insight into the immune response to EVD and its durability, with the hope of developing optimal preventative and immunotherapeutic measures for naive individuals and EVD survivors.

Multiple EBOV seroprevalence studies have been conducted across Central and Western Africa. The methodological variability ultimately complicates comparability across studies [9–18]. Few of these studies investigated EBOV-specific neutralizing antibody in a large cohort past 6 months and only 1 past 1.5 years (n = 117) [6]. Two large cohort studies in Liberia and Guinea showed high anti-EBOV glycoprotein (GP)−immunoglobulin G (IgG) seropositivity (87.2% and 98.2%, respectively) in EVD survivors approximately 1 year after resolution of symptoms. In the Guinean study, these responses waned to 76.2% over the 5-year study period [16, 18]. The seroprevalence of EVD survivor household contacts (HCs) is variable, ranging from 1.4% to 45.8% [9, 10, 14–16] with the majority of these studies determining seropositivity to be <12%. Taken together, there is a need for increased understanding of the humoral immune response during EBOV infection and the durability of humoral immunity.

In this study, we evaluate humoral responses to EBOV infection in a cohort of EVD survivors and their HCs in Eastern Sierra Leone by determining IgG seropositivity and assessing neutralization potential as a measure of antibody function. Through inclusion of HCs, we were able to determine the prevalence of subclinical infection, thereby improving our understanding of EBOV transmission in this population. Finally, we associated EBOV seropositivity and neutralization capacity with discrete symptoms and subphenotypes of post–Ebola syndrome (PES). Results from this study broaden our understanding of humoral immunity and will aid our understanding of it after vaccination and immunotherapy.

## METHODS

### Ethics Statement

This study was approved by the Sierra Leone Ethics Scientific Research Committee and the Tulane University Institutional Review Board (protocol 15-701226). All study staff were trained in responsible conduct of research concerning human subjects. The Sierra Leonean research site, Kenema Government Hospital, oversaw all informed consenting activities and interaction with participants throughout the study. All study participants provided written informed consent.

### Study Design and Data Collection

EVD survivors residing in the Eastern Province of Sierra Leone were identified through the Sierra Leone Association of EVD Survivors (SLAES) as previously described [5]. Survivors were eligible by history of EVD, inclusion on the SLAES survivor registry, and age ≥6 years. Up to 3 HCs identified by each survivor were enrolled. HCs had no history of EVD, were not present on the SLAES registry, and were ≥6 years old. At the time of enrollment, participants provided a full medical history, self-reported symptom questionnaire, and blood specimen. Whole blood was processed according to standard methods, and serum was frozen and stored for further analysis. A physical examination was performed by the lead study physician, according to standard clinical practice.

### EBOV Antibody Assessment

#### Anti-EBOV GP and VP40 Enzyme-Linked Immunosorbent Assays

Recombinant anti-EBOV enzyme-linked immunosorbent assays (ELISAs) were used for IgG detection. Samples were assayed on GP- and VP40-coated IgG ELISA detection plates (Zalgen LLC) according to the manufacturer's instructions (supplementary methods). Negative cutoffs were determined by a receiver operator curve method. Samples were considered positive if the resultant concentration was above the calculated cutoff of 7.74 and 9.99 U/mL for GP and VP40, respectively.

#### EBOV GP Pseudovirus Neutralization Assay

Pseudovirus was generated by EBOV GP (MakonaΔmuc) in an HIVΔENV (SG3) backbone. The pseudovirus was produced according to previously described methods for HIV and Lassa virus (supplementary methods) [19, 20]. Samples with >50% neutralization were considered neutralizing and further classified as weak (50% to <65%), moderate (65% to <80%), or strong (≥80%) neutralizers.

#### Assessment of Nonspecific Binding and Anti-EBOV Avidity

A nonspecific binding assay was performed on uncoated plates to rule out nonspecific binding. An avidity assay was adapted from standard methods (supplementary methods) [21, 22]. Samples were assayed as described with or without the application of 2M urea. Samples with higher binding retain high levels of reactivity despite the urea wash step. Samples were categorized by the avidity index and defined as low (<0.6), moderate (0.6–0.7), and high (>0.7) avidity.

### Statistical Analysis

Statistical analyses were performed with SAS version 9.4 (SAS Institute) and Prism version 9 (GraphPad). Analysis of demographic variables comparing survivors and HCs was conducted by logistic regression controlling for age and sex by including those variables in the model. Chi-square and *t* test analyses were performed for categorical and continuous variables, respectively. Two-sided *P* values based on a Fisher exact test were used for all 2 × 2 chi-square analyses. Chi-square and analysis of variance were used for analyses with >2 categories. Four-parameter logistic regression was used to determine calculated concentrations for anti-EBOV IgG. Symptom analyses were done with multiple logistic regression controlling for age and sex. Adjusted *P* values were generated for the symptom analyses based on the number of observations per organ system group (ie, the musculoskeletal group has 8 variables, yielding an adjusted alpha of .006 [0.05/8]). All reported *P* values were controlled for age and gender.

## RESULTS

### Demographics and Participant Characteristics

**Figure 1.**
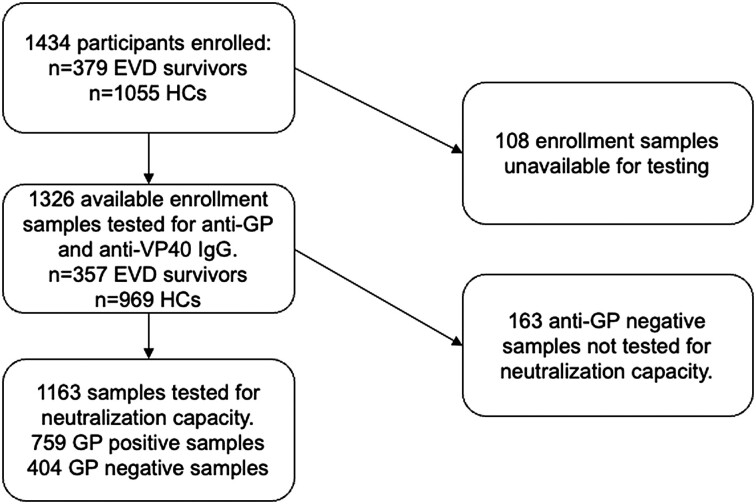
Sample testing flowchart. Participants enrolled and testing strategy are shown. EVD, Ebola virus disease; GP, glycoprotein; HC, household contact.

In total, 379 EVD survivors and 1055 HCs were enrolled between 2016 and 2019 (Figure 1) with 1326 samples available for testing: 357 EVD survivors and 969 HCs. EVD survivors were significantly older than their HCs, with mean ages of 29.8 years (range, 7–88) and 23.0 years (range, 7–75; *P* < .001; Table 1). There was no significant difference in district of origin between survivors and contacts or in sex between the comparison groups after controlling for age. The median duration of hospital stay for acute EVD was 22 days, while the median time from discharge to enrollment was 932 days, approximately 2.5 years.

**Table 1.** Participant Demographics and Characteristics

	Survivors (n = 357)	Contact (n = 969)	*P* Value
Age, y, mean (SD)	29.79 (14.48)	23.00 (12.35)	<.001
Female	195 (54.62)	454 (46.85)	.183
Age, y			
<15 (n = 135)	35 (61.40)	100 (43.10)	
15–40 (n = 429)	120 (53.33)	309 (47.54)	
> 40 (n = 85)	40 (53.33)	45 (51.72)	
Marital status (>15 y)			.003
Married	156 (61.18)	247 (68.42)	
Single	59 (23.14)	113 (31.30)	
Widowed	40 (15.69)	1 (0.28)	
Education			<.001
None	80 (31.37)	47 (7.44)	
Some primary	11 (4.31)	91 (14.40)	
Completed primary	45 (17.65)	97 (15.35)	
Some secondary	95 (37.25)	347 (54.91)	
Completed secondary	4 (1.57)	25 (3.96)	
Beyond secondary	20 (7.84)	25 (3.96)	
District of origin			…
Kenema	134 (37.54)	332 (34.26)	
Kailahun	154 (43.14)	432 (44.58)	
Kono	69 (19.33)	203 (20.95)	
Case characteristics, median (IQR)			
Hospitalization length	22 (14–30)		
Time from discharge to enrollment	932 (585–1191)		
Household contact characteristic			
Relationship to survivor			…
Live with survivor		801 (60.41)	
Caretaker of survivor		614 (46.30)	
Sexual contact		27 (2.04)	
Other		77 (5.81)	
When did you contact the survivor			…
Before illness		663 (50.00)	
During illness		614 (46.30)	
After illness		56 (4.22)	

Age, sex, marital status, and education were assessed in the survivor and contact cohorts. Statistical analysis was performed with conditional logistic regression, controlling for age and sex. Data are presented as No. (%) unless noted otherwise.

### EBOV Humoral Immune Responses

#### Anti-EBOV IgG Antibody in EVD Survivors and Contacts

**Figure 2.**
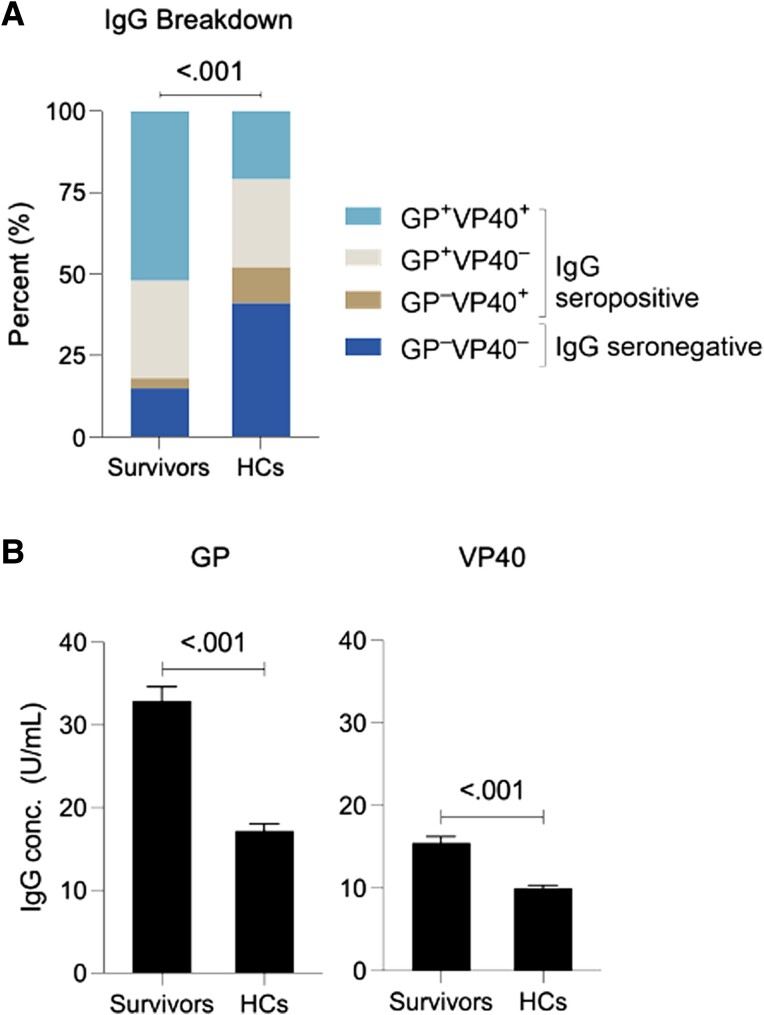
EBOV IgG concentration and seropositivity. *A*, IgG seropositivity breakdown between GP and VP40. *B*, EBOV IgG calculated concentration in survivors and HCs. Mean ± SE calculated concentration of GP and VP40 IgG in survivors (n = 357) and their HCs (n = 970, *P* < .001). Enzyme-linked immunosorbent assays were run on Zalgen IgG plates with GP calibrators developed in-house and VP40 calibrators that were developed by the manufacturer. EBOV, Ebola virus; GP, glycoprotein; HC, household contact.

All samples were screened for anti-EBOV IgG by ELISA. Significantly more survivors than the HCs were seropositive for anti-EBOV IgG (85.2% vs 59.4%, *P* < .001; Table 2). Anti-GP IgG responses dominated, particularly in survivors, with a small percentage of individuals maintaining a detectable VP40 response alone (3.1%; Figure 2*A*). In total 82.1% of survivors were anti-GP seropositive while a sizable but smaller percentage (55.2%) were anti-VP40 seropositive. A similar but less pronounced pattern was observed in HCs, with 48.8% anti-GP positive and 32.2% anti-VP40 positive. After controlling for age and sex, the mean IgG concentration was significantly higher in EVD survivors as compared with HCs for anti-GP (32.9 and 17.2 U/mL, *P* < .001) and anti-VP40 (15.5 and 10.0 U/mL, *P* < .001; Figure 2*B*, Supplementary Figure 4). Of survivors with detectable responses, 52.1% were positive for anti-GP and VP40 IgG (double positive), whereas 21.6% of IgG-positive contacts were double positive.

**Table 2.** EBOV IgG Concentration and Seropositivity in Survivors and Contacts

	Survivors (n = 357)	HCs (n = 969)	*P* Value^a^
IgG seropositive, No. (%)			
Anti-GP	293 (82.07)	473 (48.76)	<.001
Anti-VP40	197 (55.18)	312 (32.2)	<.001
Overall	304 (85.15)	576 (59.38)	<.001
IgG seropositivity breakdown,^b^ No. (%)			<.001^c^
GP+VP40+	186 (52.10)	209 (21.55)	
GP+VP40-	107 (29.97)	264 (27.22)	
GP–VP40+	11 (3.08)	103 (10.62)	
GP–VP40–	53 (14.85)	394 (40.62)	
IgG concentration, U/mL, mean (SD)			
Anti-GP	32.89 (32.68)	17.20 (26.68)	<.001^d^
Anti-VP40	15.48 (14.34)	9.97 (11.40)	<.001^d^

Abbreviations: EBOV, Ebola virus; GP, glycoprotein; HC, household contact.

^a^Chi-square.

^b^EBOV IgG concentration breakdown shows seropositivity to GP and VP40 (GP+VP40+), GP alone (GP+VP40−), VP40 alone (GP–VP40+), and EBOV seronegative (GP–VP40−) as measured by enzyme-linked immunosorbent assay.

^c^One-way analysis of variance.

^d^ *T* test.

#### Neutralization Potential of Survivors and HCs

**Figure 3.**
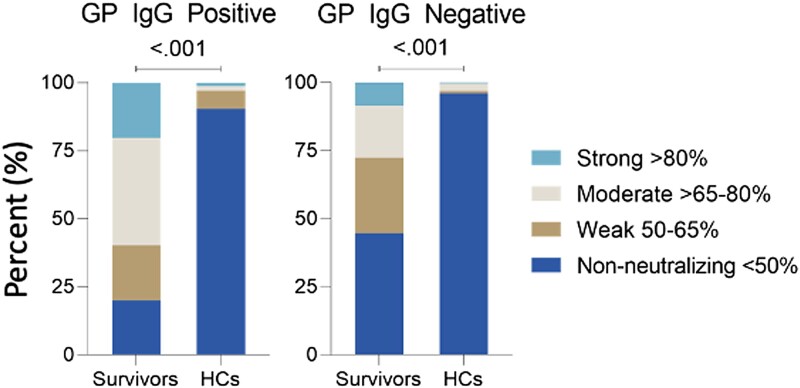
Anti-EBOV GP neutralization efficiency and durability. *A*, Percentage neutralization for participants who were EBOV GP+ and GP–. Statistical analyses were performed by analysis of variance, followed by pairwise comparisons with chi-square. *P < .05. EBOV, Ebola virus; GP, glycoprotein; HC, household contact.

To better understand anti-EBOV antibody function in our cohort, we assessed the ability of participant sera to neutralize a pseudovirus expressing EBOV GP. Neutralization assays were performed on all participants who were GP positive (n = 759) and a subset of those who were GP negative (n = 404). The majority of survivors who were GP positive (80.1%) neutralized EBOV GP pseudovirus (Table 3, Figure 3, Supplementary Figure 5). Most survivors mounted moderate (65% to <80%) neutralization responses (39.5%), whereas just over 20% of survivors mounted strong responses (≥80% neutralization). Of the survivors who were GP negative, 55.3% (26/47) demonstrated neutralization activity. In contrast to EVD survivors, 90.4% of HCs who were GP positive showed no neutralization activity.

**Table 3.** α-EBOV GP Neutralization Efficiency in EVD Survivors and Their Contacts

	Survivors (n = 338)	HCs (n = 825)	*P* Value
EBOV GP IgG positive			<.001
Nonneutralizing	58 (19.93)	423 (90.38)	
Weak	59 (20.27)	31 (6.62)	
Moderate	115 (39.52)	8 (1.71)	
Strong	59 (20.27)	6 (1.28)	
EBOV GP IgG negative			<.001
Nonneutralizing	21 (44.68)	343 (96.08)	
Weak	13 (27.66)	3 (0.84)	
Moderate	9 (19.15)	10 (2.8)	
Strong	4 (8.51)	1 (0.28)	
Overall			
Neutralizing	259 (76.63)	59 (7.15)	<.001

α-EBOV GP neutralization efficiency in EVD survivors and HCs. Neutralization responses categorized as nonneutralizing (<50%), weak (50%–65%), moderate (>65%–80%), or strong (>80%). Data are presented as No. (%).

Abbreviations: EBOV, Ebola virus; EVD, Ebola virus disease; GP, glycoprotein; HC, household contact.

#### Anti-EBOV Antibody Binding and Avidity

To determine whether GP IgG positivity in nonneutralizing contacts was due to nonspecific binding or low antibody avidity, samples with anti-GP IgG concentrations between 40 and 60 U/mL in 5 groups were assayed: nonneutralizing contacts (n = 59) and survivors (n = 11), along with weakly (n = 9), moderately (n = 25), and strongly (n = 9) neutralizing survivors. This IgG positivity determination was made to ensure that samples were well above the IgG negative cutoff level. First, we ruled out nonspecific binding by running the selected samples on uncoated, milk protein–blocked plates (supplementary methods). No tested samples showed reactivity, indicating that the seropositivity observed was indeed specific to the EBOV GP used to coat the plates (data not shown). Avidity testing demonstrated that HCs and survivors in the comparator groups all had high average avidity indices (>0.70; Supplementary Figure 6). However, mean avidity index was significantly lower in the HC group than all survivor groups (0.80 vs 0.92–0.98, *P* < .001).

#### PES and Humoral Immune Responses

Our group previously described PES symptoms and identified 6 symptom clusters that define PES within 3 primary phenotypes: asymptomatic and symptomatic with or without musculoskeletal involvement [5]. Thus, we set out to understand the relationship between symptoms common to PES and the humoral immune responses described earlier.

**Figure 4.**
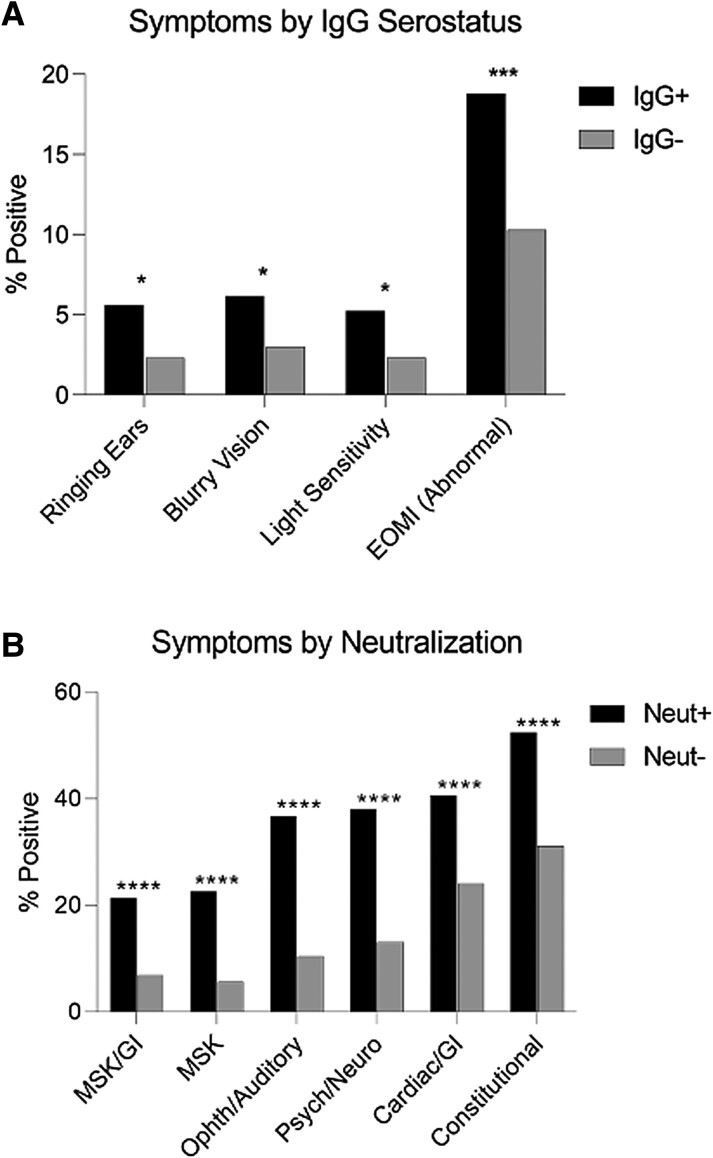
Post–Ebola syndrome symptoms and humoral immune correlates. Percentage positive for each symptom or symptom cluster by (*A*) IgG serostatus and (*B*) neutralization capacity. *P < .05. ***P < .001. ****P < .0001. EOMI, extraocular movements intact; GI, gastrointestinal; MSK, musculoskeletal.

Ophthalmologic and auditory symptoms were more common in individuals who were IgG positive vs IgG negative (blurry vision, loss of vision, abnormal extraocular movements, and ringing ears; Figure 4, Supplementary Table 2, Table 1). After adjustment for multiple comparisons, abnormal extraocular movement was the only symptom or physical examination sign significantly elevated in individuals who were IgG positive as compared with IgG negative (18.79% and 10.31%; *P* < .001). Lower extremity edema and symptoms within the cardiac/gastrointestinal cluster were more common in participants who were IgG negative (*P* < .001 and *P* = .0058). Survivors who were IgG positive were less affected by discrete cardiac and constitutional symptoms, such as heart palpitations, dizziness, night sweats, and increased urination. However, these associations failed to meet significance after adjusting *P* values for multiple comparisons (Table 2). Participants with neutralizing antibody experienced a significantly higher burden of PES symptoms vs their nonneutralizing counterparts (Table 3). There was no significant difference in identified PES phenotype (asymptomatic vs symptomatic with or without musculoskeletal involvement) in relation to overall IgG serostatus, serostatus within survivors alone, or neutralization.

## DISCUSSION

In this study, we describe humoral immune responses targeting EBOV GP and VP40 in EVD survivors and HCs >2 years after resolution of acute EVD. We found high levels of seroprevalence in survivors, with a surprisingly high proportion of HCs presenting with a measurable antibody response. The neutralizing antibody response in survivors was mainly moderate to strong, whereas >90% of EBOV GP-positive HC samples demonstrated no neutralizing activity. Variable immune responses in survivors and HCs with evidence of exposure suggest a spectrum in the quality of humoral immune responses.

EBOV GP is a primary target of survivor humoral immune responses and an important contributor to cytotoxicity and EVD pathogenesis [16, 23–25]. We found that 82.1% of survivors maintained detectable antibodies to EBOV GP 2.5 years after disease resolution. Similar reports from Liberia and Guinea cited 87.2% of anti-GP seropositivity in EVD survivors within 1 year and 96.3% within 2 years of convalescence, respectively [16, 18]. In addition, 56.2% of survivors in our cohort developed anti-VP40 responses, lower than the 95.0% (n = 687) of Guinean survivors [18]. Antibodies to VP40 were most often observed in combination with anti-GP IgG, with only 3.1% of survivors having detectable VP40 antibodies alone. Two studies reported that 100% of survivors enrolled (n = 4 and n = 7) developed anti-VP40 responses within 6 months of convalescence [26, 27]. However, others indicated that 21% of survivors (n = 14) had sustained anti-VP40 responses 40 years after recovery from disease and all were also anti-GP reactive, suggesting that antibodies directed at VP40 are not as long-lasting as those to GP [24]. Diallo et al showed a more rapid waning of VP40 antibodies than GP antibodies over a 5-year study period [18].

It is likely that the 14.9% of survivors lacking anti-GP and-VP40 responses in our cohort developed responses to GP and VP40 that have since waned, as seen in other studies [8, 18], or generated antibody responses to other proteins as opposed to demonstrating an absence of a humoral immune response altogether. Stronger antibody responses to EBOV NP vs VP40 have been documented by several studies, making NP a likely alternative protein target in survivors lacking GP and VP40 responses [18, 24, 26, 27]. Additionally, the sacrifice of sensitivity for specificity in our positive cutoff may have contributed to lower rates of anti-GP IgG-positive survivors than those in Liberia [16]. Due to known circulation of other filoviruses within Sierra Leone, higher specificity was considered critical [11, 12]. Assay differences in sensitivity and specificity may largely explain differences in observed results across studies, warranting future head-to-head comparisons. Finally, there is a possibility that a subset of EVD survivors were misdiagnosed with EVD.

In addition to examining survivors, we investigated anti-EBOV seropositivity in HCs to better understand potential asymptomatic or subclinical infections. Widely varied rates of asymptomatic infection with EBOV have been documented [10, 14, 16, 28]. One study found a dose-dependent relationship between EBOV infection and development of symptomatic disease [29]. Here we report 59.4% of HCs to be anti-GP IgG positive, anti-VP40 positive, or both, with 48.8% of HCs anti-GP IgG positive. This rate is higher than previous studies, with the majority determining HC seroprevalence to be <12%, and it is higher than asymptomatic infection rates presented by a meta-analysis that estimated secondary infection rates between 15.4% and 22.9% in HCs [9, 14–16, 30]. This finding, in addition to previous studies on asymptomatic infection rates, indicates a higher potential transmission rate than previously recorded, warranting further study of EVD epidemiology and transmission dynamics. Additionally, our cohort is from a region of Sierra Leone that was first affected by the Ebola outbreak during a period when community engagement, contact tracing, and case-finding efforts were in their infancy. Thus, it is probable that during these outbreak conditions, those with mild or moderate symptoms could have been dissuaded from seeking care at an Ebola treatment unit due to fear and misinformation. Finally, the previously recorded presence of filovirus antibodies in Sierra Leone prior to 2013 with the identification of Bombali virus in bats native to Sierra Leone suggests that some of the detected EBOV antibodies may be due to exposure to another related filovirus [11, 31]. It is possible that the seropositivity to multiple EBOV antibodies observed in approximately 20% of our HC cohort indicates a greater specificity to EBOV infection. However, further studies on EBOV cross-reactivity must be done in this context to answer that question definitively.

We characterized antibody function in survivors and HCs using a pseudovirus neutralization assay. We found that 76.6% of survivors tested were able to neutralize ≥50% of the virus, 17.6% of whom demonstrated strong neutralizing activity (>80%). Of the survivors who were anti-GP IgG positive, 80.1% of participants were able to neutralize >50% of the virus. Previous reports have indicated that neutralizing antibodies following EVD can be particularly long-lasting, with 28% of survivors from the 1976 outbreak capable of neutralizing >50% of virus 40 years after infection [24]. Among other explanations, it is likely that IgG-positive nonneutralizers developed neutralizing antibody that has since waned or their antibodies have another Fc-mediated functional profile in addition to neutralization. A study investigating antibody functional profiles in survivors of EVD demonstrated that antibodies that confer high levels of complement activation and moderate levels of cytotoxic cell activation are highly protective in a mouse model, with 100% (n = 5) of mice surviving EBOV infection [32]. These data suggest that antibody function beyond neutralization may contribute to reduced mortality in the context of EVD; however, nonneutralizing antibody responses must be studied further in animal models and humans to fully understand their role in protection from fatal EBOV infection. In contrast, the detection of neutralizing antibody in IgG-negative samples suggests that antibodies with neutralizing potential were present at levels below the determined cutoff or targeted quaternary epitopes not present in the recombinant proteins of the ELISA.

It is notable that 9.61% and 3.92% of HCs who were IgG positive and negative in our study, respectively, had EBOV-specific neutralizing antibody. This finding, with recent data from EVD survivor studies in Guinea showing detection of neutralizing antibodies from HCs [6], provides strong support for the concept that subclinical infections occur and should be investigated further to understand the role of these individuals in EBOV transmission. We found that among 48.8% of HCs with detectable anti-GP IgG, only 9.6% had neutralizing antibodies; this finding suggests that the antibodies present in some contacts may be cross-reactive from another infection or nonfunctional or they might possess another Fc-mediated function. Our antibody avidity experiments showed that GP IgG–positive nonneutralizing HCs had antibody with relatively high avidity, thus ruling out low antibody avidity as the cause of the lack on neutralizing antibody. Further investigation beyond the scope of this article is in process, including longitudinal studies and innate antibody function, which will shed light on antibody function in the HC cohort over time.

To gain a better understanding of sequelae experienced following EVD, we analyzed symptoms experienced at enrollment as compared with measured humoral immune responses. We found that ophthalmologic and auditory symptoms were more common in participants who were IgG positive vs IgG negative overall and that survivors who were IgG positive were more likely to present with constitutional symptoms than those who were IgG negative. Individuals with neutralizing antibody experienced a much higher burden of PES symptoms than nonneutralizers. The mechanisms underlying PES are not well understood; neutralizing antibody could be associated with the production of autoantibodies, as recently described in “long COVID” [33]. Further investigation is needed to understand mechanisms driving relationships among EBOV IgG seropositivity, neutralization capacity, and PES symptoms.

### Study Limitations

There are several limitations that must be considered when interpreting the results of this study, primarily surrounding the elevated EBOV seropositivity levels that we identified in the HC cohort as compared with previous studies, which brings the question of false-positive risk. To be confident in our ELISA results and to reduce the potential for false-positive risk, we developed objective assay cutoffs using a comprehensive panel of known positive (n = 370) and negative (n = 200) samples collected from the region prior to 2013. This strategy utilizing regional controls was modeled after previously published methods [34] and is detailed in the supplemental methods. Additionally, unexposed negatives were run on each assay plate, which showed no reactivity on any of the plates run. Differences in performance of the testing platform that we used vs those used in previous studies could also explain the discrepant results. We could not directly control for differences in assay sensitivity and specificity between assays because a head-to-head comparison of the assay used here with previously reported assays has not been done and is beyond the scope of this article.

Another major consideration is potential misclassification of EVD survivors and controls, which could lead to biases in the results. We were unable to differentiate between cases and controls based on symptomology or EBOV diagnostic tests during acute infection, as we had no information collected on acute illness during the outbreak. To reduce the introduction of recall bias, we chose not to rely on participant recollection about illness during the EVD outbreak. We based inclusion on record of discharge from an Ebola treatment unit after a positive EOBV test result. We believe that the surprisingly high seropositivity levels in the HC cohort points to unrecognized transmission. This is an important point considering the accepted severe case definition of acute EVD and, if true, further complicates EVD case identification. These considerations must be kept in mind when responding to future outbreaks and likely call for greater levels of testing of asymptomatic or mildly symptomatic contacts in future outbreaks.

## CONCLUSIONS

This study contributes to the growing body of knowledge on humoral immunity in EVD survivors. Here we have shown that most survivors develop sustained antibody responses to EBOV GP and to a lesser degree VP40. Additionally, strong neutralization capacity does not seem to be sustained in all EVD survivors, and this calls for further study of sustained T cell–mediated immunity or nonneutralizing Fc-mediated innate effector functions, which may also play an important role. Finally, we have identified evidence of seropositivity to EBOV in HCs at a higher rate than previously reported. These data suggest that asymptomatic or paucisymptomatic infection could occur at higher rates than expected or that cross-reactive pathogens may be more prevalent than expected, requiring further study. Taken together, these data provide insight of humoral responses and will contribute to future therapeutic and vaccine development.

## Supplementary Data

Supplementary materials are available at *The Journal of Infectious Diseases* online (http://jid.oxfordjournals.org/). Supplementary materials consist of data provided by the author that are published to benefit the reader. The posted materials are not copyedited. The contents of all supplementary data are the sole responsibility of the authors. Questions or messages regarding errors should be addressed to the author.

## Supplementary Material

jiae155_Supplementary_Data

## Notes

***Acknowledgments.*** We acknowledge the Sierra Leone Association of Ebola Survivors and all of the participants in this study.

***Author contributions.*** Conceptualization: J. S. S., D. A. W., W. A. F., D. S. G. Methodology: J. S. S., D. S. G. Formal analysis: N. G. B., K. R. S. Investigation: N. G. B., K. R. S., E. E. C., M. A. G., L. K., I. M. K., F. K. K., M. M., J. D. S., D. E., A. R. S., S. C. F., R. J. S. Resources: J. E. R. Data curation: J. G. S., E. J. E., F. A-H. Writing–original draft: N. G. B., J. S. S. Writing–review and editing: N. G. B., K. R. S., E. E. C., D. A. W., R. F. G., J. S. S. Visualization: N. G. B. Supervision: J. S. S. Project administration: J. S. S., D. S. G. Funding acquisition: J. S. S., D. A. W., W. A. F., R. F. G.

***Financial support*.** This work was supported by the National Institutes of Health (R01 AI123535 to W. A. F., D. A. W., J. S. S.); and the West African Research Network for Infectious Diseases (WARN-ID) (National Institutes of Health U01 5U01AI151812 to R. F. G., J. S. S., N. G. B., E. J. E.).
