# Supplementary material for: Ebola Virus–Specific Neutralizing Antibody Persists at High Levels in Survivors 2 Years After Resolution of Disease in a Sierra Leonean Cohort
Source: J Infect Dis. 2024 May 27;230(4):e929–37. doi: 10.1093/infdis/jiae155 (PMC11481455; doi:10.1093/infdis/jiae155)
Supplement: jiae155_Supplementary_Data [file jiae155_supplementary_data.zip › R1JID-77264_EBOV IgG Supplemental.docx]

Supplemental Methods

*Anti-GP ELISA Calibrator Development*. We used pre-coated, pre-blocked plates purchased from Zalgen Labs, LLC (Germantown, MD). Calibrators for the GP IgG ELISA plates were developed to standardize results across plates and to allow interpolation of unknown values from test samples. Ten EVD survivor sera highly responsive to an in-house screening ELISA and with high neutralization titers were titrated on a GP plate, beginning with an initial dilution of 1:100. anti-GP monoclonal antibodies (J Robinson, personal communication) were used as positive controls for plate performance. The eight sera with the strongest signal were selected and pooled in equal volumes to create a positive control calibrator for the anti-GP plates (Supplemental Figure 1). Reactivity of the pooled sera calibrator was confirmed through further testing on GP ELISA plates. These calibrators were then used to determine relative units per milliliter (U/mL) of GP concentration to standardize ELISA results across experiments.

*Anti-EBOV GP and VP40 ELISAs.* Samples were assayed according to manufacturer’s instructions. Briefly, samples and assay controls were diluted 1:100 in sample diluent. For VP40 plates, a 5-point reference curve using 3-fold serial dilutions was prepared using provided calibrators. For GP plates, calibrators were developed and diluted 1:100 in sample diluent, followed by a 6-point reference curve using 3-fold serial dilutions (see Supplemental Methods). All controls and samples were plated in duplicate and incubated at room temperature. Un-exposed negative controls were run on each plate. Following the initial 30-minute incubation, plates were washed (Biotek, 405Select) five times in provided wash buffer. Plates were incubated in IgG anti-human HRP-conjugated antibody solution for 30 minutes, followed by another wash step. The one-component substrate was then added, and plates were allowed to incubate at room temperature for 15 minutes before the addition of stop solution. Plates were read at 450 nm (Biotek, Synergy H1).

*Negative Cutoff Calculation*. Samples collected prior to 2013 from both Sierra Leone by our group and Nigeria (provided by Pardis Sabeti as part of the Viral Hemorrhagic Fever Consortium) were used to determine negative cutoffs. Sierra Leonean samples (n=100) were collected as part of a Lassa fever serosurvey conducted in the region in 2007-2008 and used as healthy controls for that study. Nigerian controls (n=100) were collected near Irrua, Nigeria in 2011 as part of a genome-wide association study of Lassa fever in a Lassa endemic region. The samples collected from these individuals were used as population controls and reported never having any VHF-like symptoms. Positive control samples were collected from know Ebola survivors from Sierra Leone (n=370).

Negative cut-offs were determined using receiver operating characteristic (ROC) curve analysis, a well-established method of determining cutoff values for ELISA, and a control sampling strategy based on the Filovirus Animal Non-Clinical Group anti-GP IgG ELISA assay development (1). All control samples described above were assessed by GP and VP40 ELISA (Zalgen Labs, LLC, Germantown, MD). The top 95th percentile of both the Sierra Leonean and Nigerian controls was excluded from further analysis. The groups were combined and data input into the ROC curve along with known EVD survivor data as a positive control (Supplemental Figure 2). Optimal negative cutoffs were determined to be 7.74 U/mL (95% specificity and 82.8% sensitivity) for the GP plates, and 9.99U/mL (94.9% specificity and 55.2% sensitivity) for the VP40 plates (Supplemental Table 1). Specificity was prioritized in determining cutoffs to reduce type-I error and thus the rate of false positive determinations. Sample interpolation and ROC curve analysis were conducted in GraphPad/PRISM version 9 (San Diego, CA, USA).

*EBOV GP Pseudovirus Neutralization Assay.* The EBOV GP pseudovirus was titrated in TZM cells to determine appropriate dilution, with target luminescence of 10,000 RLU. Sera were diluted 1:100 in DM10 (DMEM + 10% FBS) and incubated with pseudotype virus at 37⁰C for one hour. Adherent TZM cells were trypsinized, prepared in DM10 and plated at 50,000 cells per well. The mixture was incubated at 37⁰C for 48 hours. Following incubation, 150μL media was removed and the mix was incubated with Bright-Glo (Promega) diluted 1:6 in lysis buffer for two minutes. Luminescence was measured and neutralization percentages were calculated for each sample using the following equation:

$$N=1-\left( \frac{{Lum}_{sera}}{{Lum}_{virus}} \right)$$

Where N=percent neutralization, Lum_sera_=luminescence reading of sample well including virus and serum, and Lum_virus_=luminescence reading of the control well with virus alone.

*Non-specific binding assessment.* An initial screen for non-specific binding was performed by running samples on uncoated plates. Plates were mock coated with 0.1M sodium bicarbonate (Fisher Chemical; Waltham, MA) and incubated for 30 minutes at room temperature. Plates were then washed with 1xPBS+0.5%Tween and blocked with 1xPBS+0.5%Tween+5%dry milk+4%whey+10%FBS for 30 minutes at 37^o^C. Samples were then loaded onto plates and the remainder of the assay followed the ELISA protocol described in the main text.

*Avidity assay*. Sample avidity was determined using parallel testing with and without a urea wash according to standard protocols. In brief, ELISAs were set up as described above. After samples were washed, either 2M urea or PBS was added and incubated for 30 minutes at room temperature. Wells were then washed, HRP was added, and the remainder of the protocol followed the ELISA method described in the main text. After reading each plate at 450nm, untreated samples were compared to those with urea to determine the avidity index for each samples using the following equation:

$AI={Conc}_{U}/Conc$

Where AI=Avidity Index, Conc_U_=Concentration with urea wash, and Conc=Concentration without urea wash.

Supplemental Figures


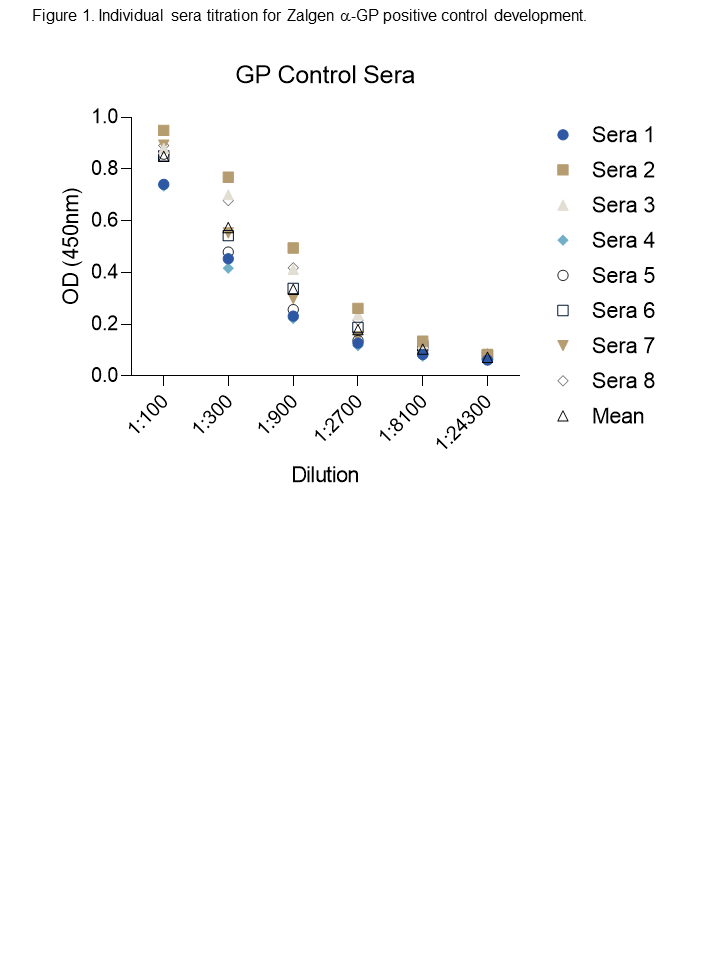


**Supplemental Figure 1. Sera titration for anti-GP positive control development.** Eight sera highly reactive to an anti-GP screening ELISA were titrated on commercial GP plates, pooled and utilized as calibrators for remaining samples.


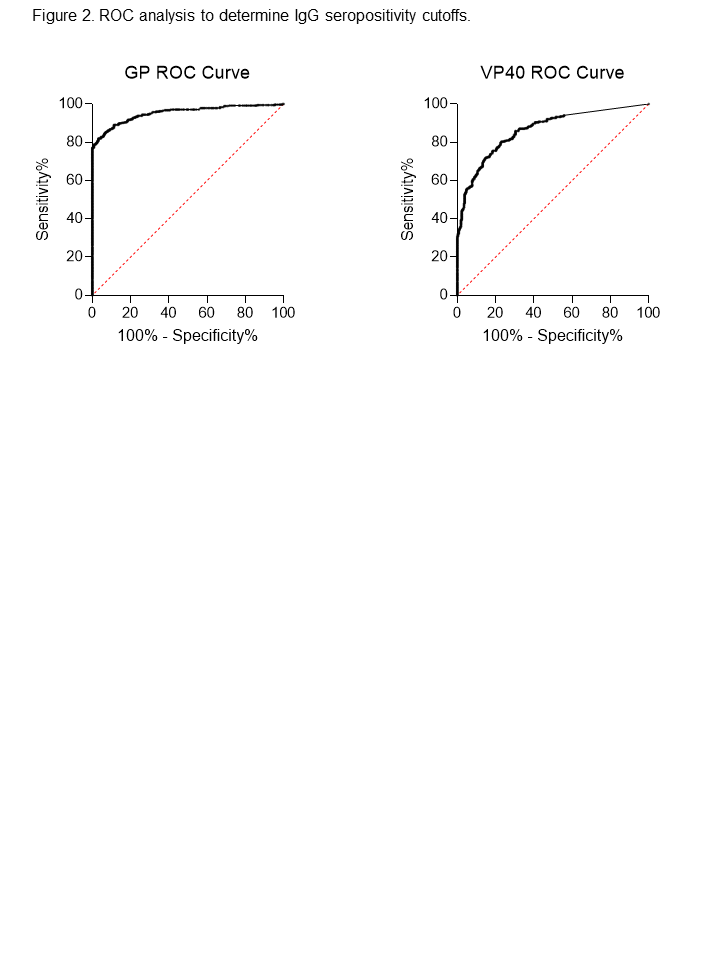
**Supplemental Figure 2. ROC analysis to determine seropositivity cutoffs.** Negative cutoffs for GP and VP40 ELISAs were determined using ROC curve analysis. Two hundred samples from West Africa collected between 2007-2011 prior to were used as negative controls, known EVD survivors were used as positive controls.

**Supplemental Table 1. ROC data for negative cutoff calculations (xlxs).** Sensitivity and specificity values for each cutoff concentration value for GP (Tab 1) and VP40 (Tab 2). Confidence intervals are presented for each sensitivity and specificity level.


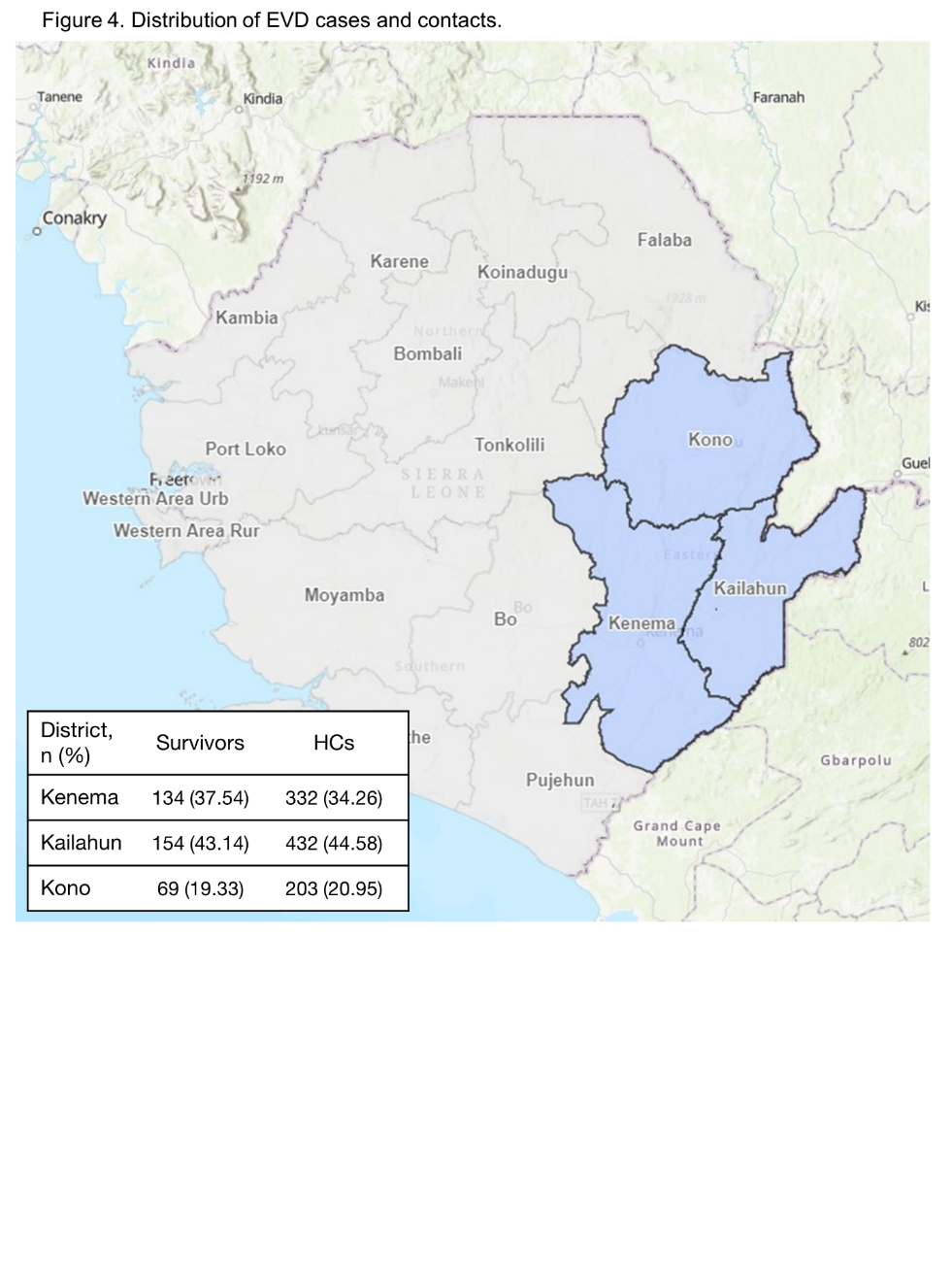


**Supplemental Figure 3. Geographical distribution of study participants.** Study subjects were enrolled in Eastern Province of Sierra Leone in Kenema, Kailahun, and Kono Districts.

**
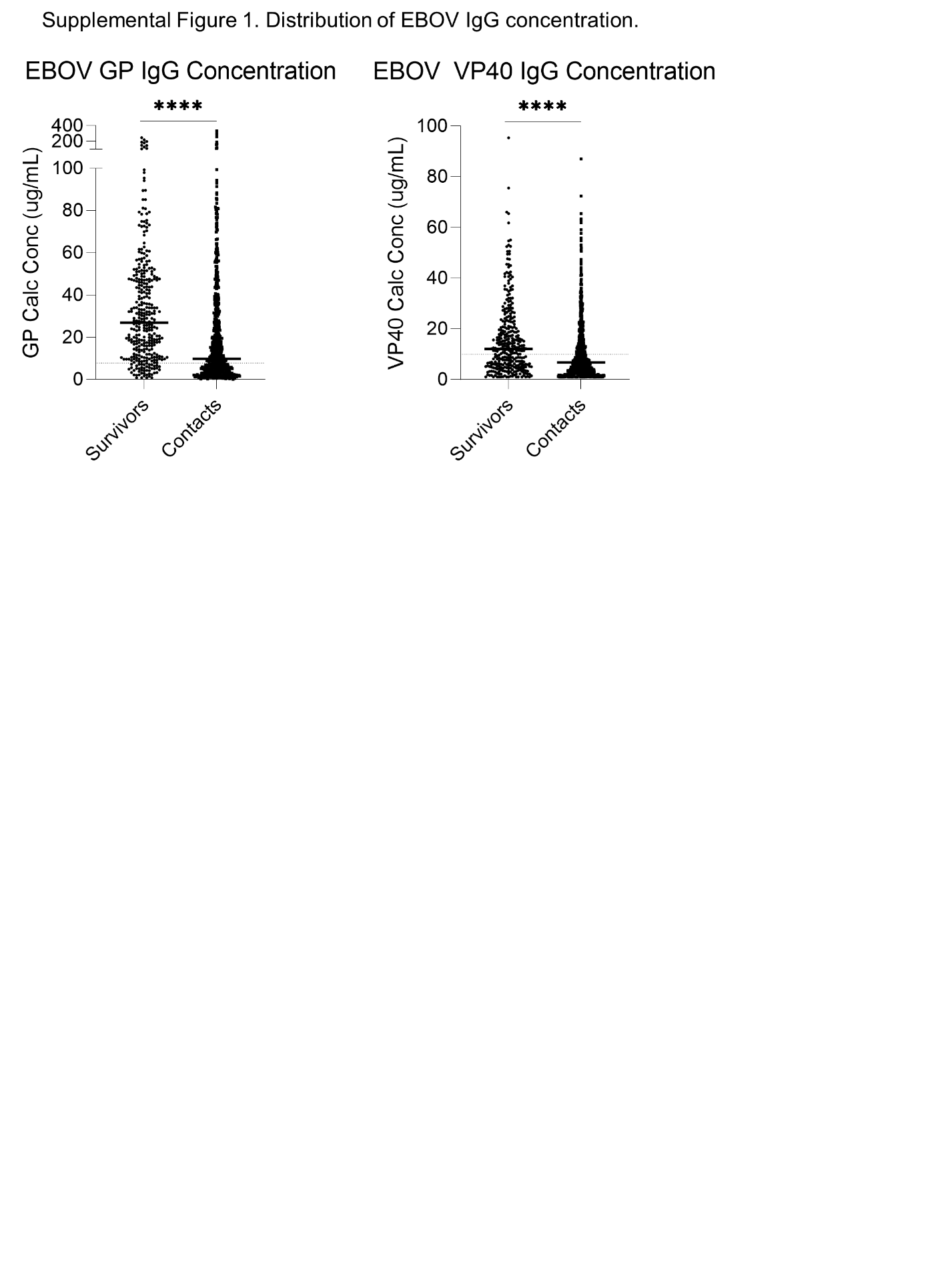
Supplemental Figure 4. Distribution of EBOV IgG Concentration.** EBOV GP and VP40 IgG concentration in EVD survivors and contacts showing the distribution of IgG positivity in each group. p<.0001 (****).

**
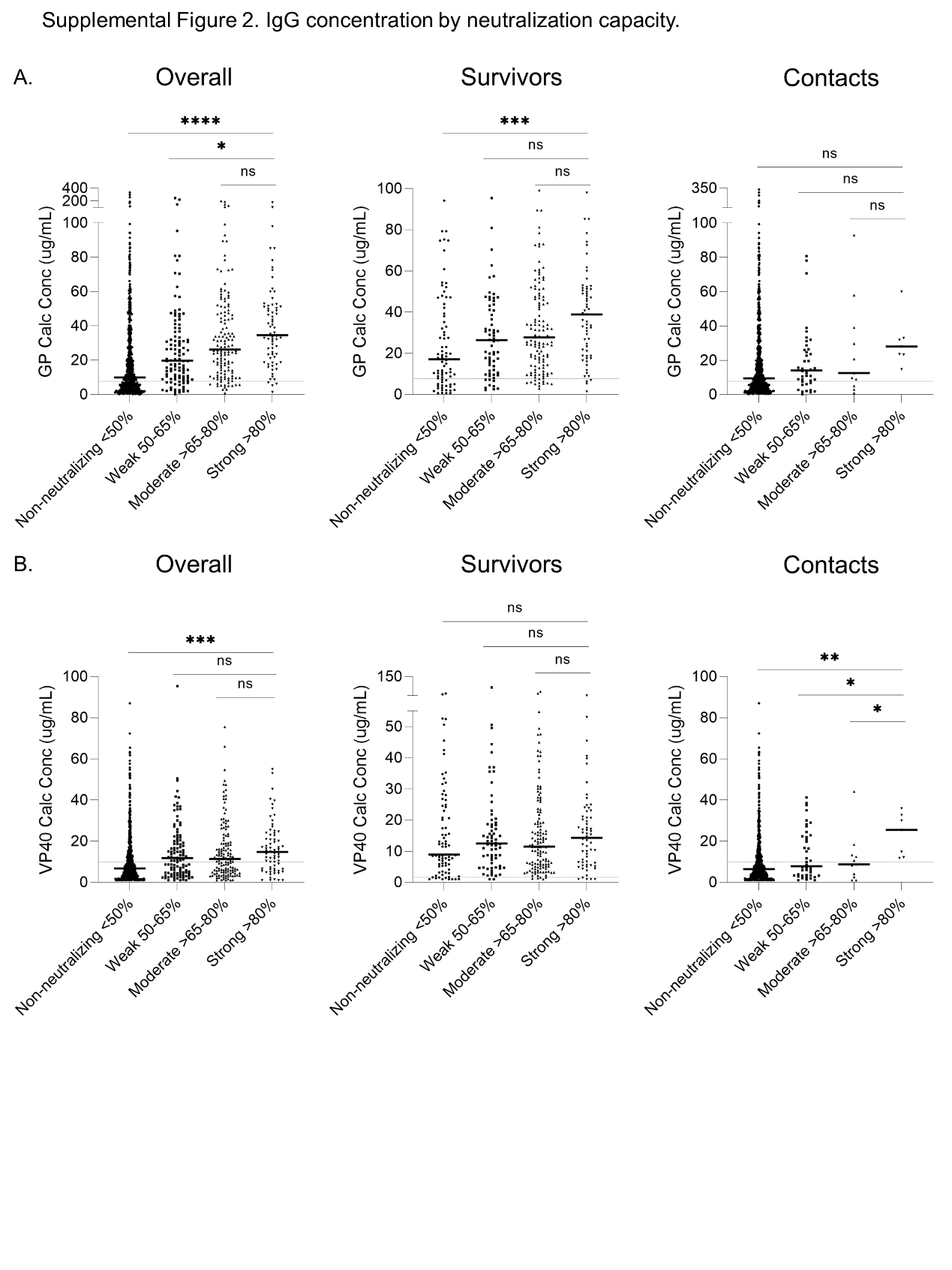
**

**Supplemental Figure 5A&B. IgG concentration by neutralization capacity.** IgG concentration is graphed along with neutralization category for GP (A) and VP40 (B) IgG. P<.05 (*), <.01 (**), <.001 (***), <.0001 (***).

**
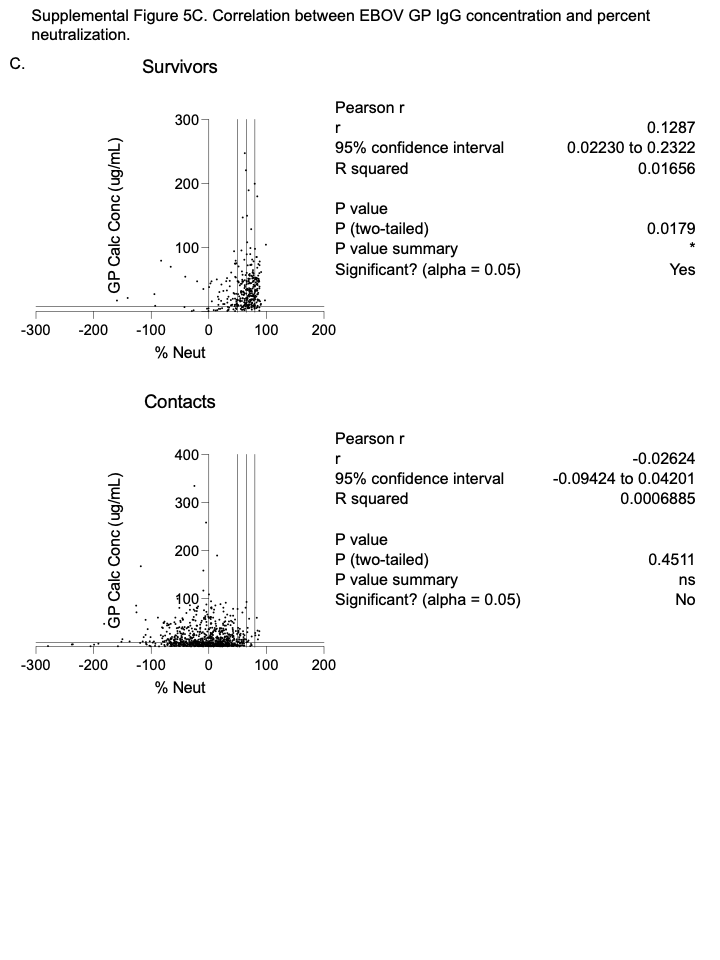
Supplemental Figure 5C. IgG concentration by neutralization capacity.** (C) Pearson’s correlations between EBOV GP IgG concentration and percent antibody neutralization are shown. Horizontal line indicates the EBOV GP IgG cutoff for seropositivity. The three vertical lines indicate the antibody neutralization categories weak (50-65%), moderate (>65%-80%) and strong (>80%).

**
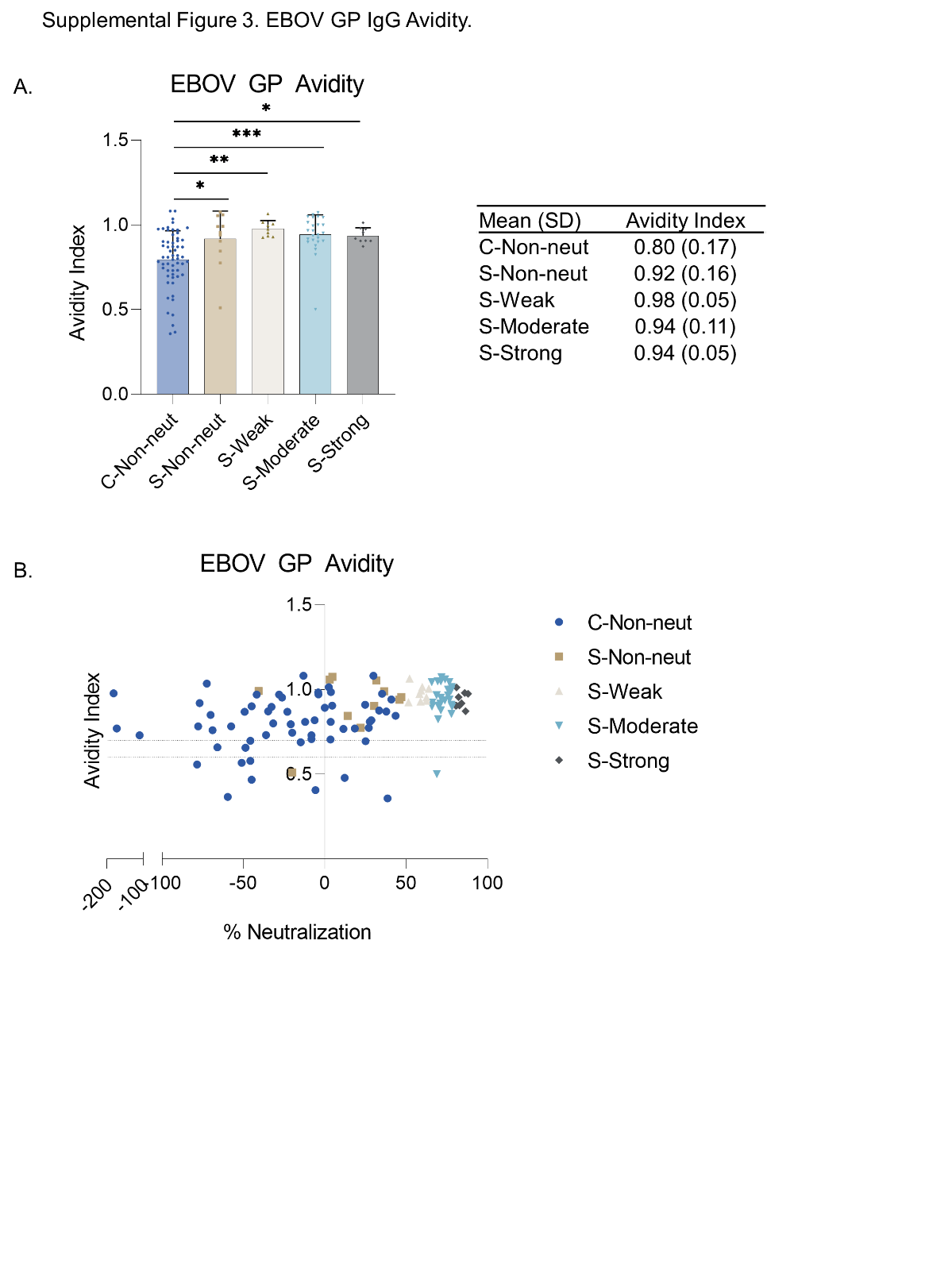
Supplemental Figure 6. EBOV GP IgG avidity.** Avidity testing was done for a subset of IgG positive individuals with GP IgG concentrations between 40-60U/mL. Avidity index is graphed with neutralization category (A) and total % neutralization (B) to show the relationship between neutralization and GP IgG avidity. Horizontal lines indicate low, moderate and high avidity. P<.05 (*), <.01 (**), <.001 (***).

**Supplemental Table 2. Post-Ebola symptoms and humoral immunity (xlxs).** Self-reported symptoms, physical exam signs, and previously identified symptom clusters were assessed with respect to overall IgG seropositivity (Tab 1), IgG seropositivity within EVD survivors (Tab 2), and neutralization capacity (Tab 3). Physical exam signs are italicized. P<.05 are in bold. Comparisons are by multiple logistic regression unless otherwise indicated. ** indicates the comparator.

1. Logue J, Tuznik K, Follmann D, Grandits G, Marchand J, Reilly C, et al. Use of the Filovirus Animal Non-Clinical Group (FANG) Ebola virus immuno-assay requires fewer study participants to power a study than the Alpha Diagnostic International assay. J Virol Methods. 2018;255:84-90.
